# Variability in organ-specific *EGFR* mutational spectra in tumour epithelium and stroma may be the biological basis for differential responses to tyrosine kinase inhibitors

**DOI:** 10.1038/sj.bjc.6602557

**Published:** 2005-04-19

**Authors:** F Weber, K Fukino, T Sawada, N Williams, K Sweet, R M Brena, C Plass, T Caldes, G L Mutter, M A Villalona-Calero, C Eng

**Affiliations:** 1 Human Cancer Genetics Program, Comprehensive Cancer Center, The Ohio State University, Columbus, OH 43210, USA; 2 Department of Molecular Virology, Immunology and Medical Genetics, College of Medicine and Public Health, The Ohio State University, Columbus, OH 43210, USA; 3 Clinical Cancer Genetics Program, Comprehensive Cancer Center, The Ohio State University, Columbus, OH 43210, USA; 4 Division of Human Genetics, Department of Internal Medicine, College of Medicine and Public Health, The Ohio State University, Columbus, OH 43210, USA; 5 Laboratory of Molecular Oncology, San Carlos University Hospital, CP 28040 Madrid, Spain; 6 Department of Pathology, Brigham and Women's Hospital, Harvard Medical School, Boston, MA 02115, USA; 7 Division of Hematology/Oncology, Department of Internal Medicine, College of Medicine and Public Health, The Ohio State University, Columbus, OH 43210, USA; 8 Cancer Research UK Human Cancer Genetics Research Group, University of Cambridge, Cambridge CB2 1XZ, UK

**Keywords:** small-molecule therapy, tyrosine kinase, EGFR mutation, breast cancer

## Abstract

Organ-specific differences in epidermal growth factor receptor (*EGFR*) mutational spectra and frequencies were found in lung cancer and sporadic and *BRCA1/2*-related breast cancers. Additionally, we found a high frequency of *EGFR* mutations in the tumour stroma of these invasive breast carcinomas. Those organ-specific mutational spectra and potential targets in the cancer-associated stroma might influence the efficacy of TKI therapy.

The most common cancers worldwide are lung and breast cancer, accounting for over 2 million new cases each year. Approximately 3 years ago, molecular targeted therapy, starting with the introduction of imatinib, which targets the tyrosine kinases (TK) BCR–ABL, KIT and PDGFR, for the treatment of chronic myeloid leukaemia, was anticipated to provide a new approach to fight malignancies. In clinical trials, the response to epidermal growth factor receptor (EGFR) inhibitors, such as gefitinib, has varied widely, ranging from rare in breast cancers to 10–30% in metastatic non-small-cell lung carcinoma (NSCLC) (Dancey and Freidlin, 2003). Recently, somatic mutations in the TK domain of the *EGFR* gene have been identified in the NSCLC of the patients who showed increased response to gefitinib, suggesting clinical utility as a predictive factor (Lynch *et al*, 2004; Paez *et al*, 2004). Despite the promising response in a subset of NSCLC, other solid tumours, such as breast cancer, do not share this. We hypothesised that differences in *EGFR* somatic mutational spectra and frequencies among the different solid tumours may result in different responsiveness. Thus, we sought to determine and compare the mutational frequencies and spectra in NSCLC and breast carcinomas. In addition, regarding the growing understanding of the tumour–stroma interaction and the possible role of cancer-associated mesenchyme as a novel target for anticancer therapy, we also analysed the stroma of invasive breast adenocarcinomas for *EGFR* mutations.

## MATERIALS AND METHODS

The TK domain of *EGFR*, encoded by exons 18–21, was directly sequenced in 60 NSCLC samples, 48 samples of sporadic breast carcinoma and 24 samples from hereditary breast cancers (17 with *BRCA1* mutations and seven with *BRCA2* mutations, respectively). All samples were obtained as anonymised archival material under approval from the respective Institutional Review Boards.

### DNA extraction and mutation analysis

In the breast cancer samples, tumour epithelial and stromal components were collected separately with laser-capture microdissection (LCM). For NSCLC, tumour-enriched paraffin-embedded samples have been used. Genomic DNA was extracted by proteinase K digestion as described by us previously (Fukino *et al*, 2004). We directly sequenced exons 18–21 of the TK domain in the *EGFR* gene with the primers listed below. PCR consisted of 40 cycles using an annealing temperature of 55°C in a 15 *μ*l reaction mixture containing 7.5 *μ*l HotStar MasterMix, 1.5 *μ*l 5xQ-Solution (Invitrogen, Carlsbad, CA, USA) and 0.25 *μ*l of each primer. PCR products were then sequenced using Big Dye v3.1 terminator technology and the ABI 3730 analyzer (Applied Biosystems, Perkin-Elmer Corp., Norwalk, CT, USA) according to the manufacturer's recommendation for mutation analysis (supplements 1 and 2).

We used two-sided Fisher exact test to evaluate differences between groups.

Primers for mutation analysis were as follows: exon 18 – GCTGAGGTGACCCTTGTCTC (sense), ACAGCTTGCAAGGACTCTGG (antisense); exon 19 – CATGTGGCACCATCTCACA (sense), CAGCTGCCAGACATGAGAAA (antisense); exon 20 – CACACTGACGTGGCCTCTCC (sense), TATCTCCCCTCCCCGTATCT (antisense); exon 21 – CCTCACAGCAGGGTCTTCTC (sense), CCTGGTGTCAGGAAAATGCT (antisense).

## RESULTS

**Figure 1:**
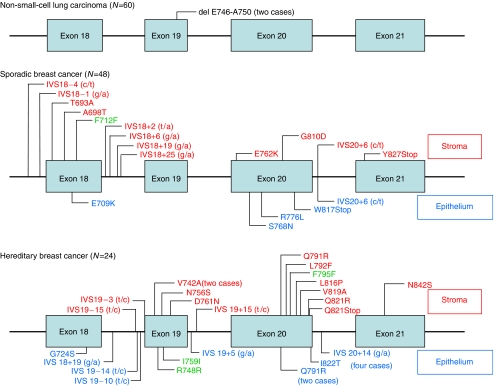
Spectra of somatic *EGFR* mutations. Location of somatic mutations found in 60 NSCLC, 48 sporadic and 24 hereditary (*BRCA1/2* mutation positive) breast cancers. Exons are shown as bars with intronic regions as lines. For breast cancer samples, labels above each bar/line indicate mutations in the stroma, and labels below denote mutations found in neoplastic epithelium. Green labels indicate silent variants.

In two (3.3%) of the 60 NSCLC samples, somatic in-frame deletions were detected in exon 19 (Table 1, Figure 1). Both samples showed a heterozygous in-frame deletion of five amino acids (E, L, R, E, A) (delE746-A750) through loss of nucleotides 2235–2249 and 2236–2250, respectively. These two deletions coincided with those reported in the previous reports, suggesting gefitinib responsiveness (Lynch *et al*, 2004; Paez *et al*, 2004).

**Figure 2:**
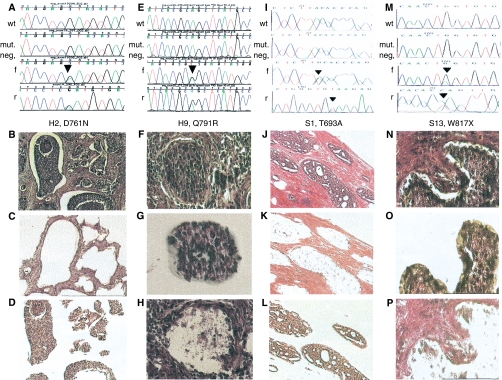
Somatic *EGFR* mutations in the epithelium or stroma of sporadic and hereditary breast carcinomas. Each of the four columns (**A**–**D**, **E**–**H**, **I**–**L** and **M**–**P**) represents one *EGFR* mutation-positive sample and the corresponding images taken during the LCM process. The sample codes corresponding to Table 1 are indicated below each set of chromatograms. Each set of chromatograms (**A**, **E**, **I** and **M**) shows the control (wild-type) sequence in the top row, followed by the sequence of the mutation-negative compartment. The heterozygous mutation and surrounding sequences are shown in forward (f) and reverse (r) directions in the bottom two rows. The first column shows sample H2, harbouring a somatic D761N mutation in the tumour epithelium (**A**, f and r) but not tumour stroma (mut. neg. in **A**). Image (**B**) shows an overview of this tumour (H&E, × 100 and × 200) and images (**C**) and (**D**) confirm that we accurately captured stroma (**C**) and epithelium (**D**). The second column shows the chromatograms (**E**) and tissue image (**F**) of sample H9, harbouring the somatic Q791R mutation in the stroma (f and r in (**E**)) but not epithelium (**E**, mut. neg.). The corresponding images (**G**) and (**H**) depict the captured epithelium (**G**) and the tissue image after extraction of the epithelial component by LCM (**H**). The third column represents the sporadic breast adenocarcinoma sample S1 (**J**) with a somatic T693A mutation in the stromal compartment (**I**, f and r) but not epithelium (mut. neg. in (**I**)). Again, images (**K**) and (**L**) verify the separation of tumour epithelium (**L**) and stroma (**K**). The last column shows sample S13 harbouring a W817X mutation in the tumour epithelium (**M**, f and r) but not stroma (**M**, mut. neg.). The neoplastic epithelium is microdissected (**O**) out of the whole tumour section (**N**), leaving the stromal compartment (**P**).

In total, seven somatic missense mutations were detected in seven (14.6%) of 48 sporadic breast cancer samples. No correlation was detected between tumour grade and mutation status. We identified 14 missense mutations in 11 (45.8%) of 24 breast cancers from *BRCA1/2* mutation carriers (Figures 1 and 2, Table 1). Thus, the frequency of *EGFR* mutations was significantly higher in *BRCA1/2-*related breast cancers compared to that in sporadic ones (*P*=0.0079). In addition, three silent mutations that did not alter the amino acid were identified in three hereditary breast cancer samples, of which two also harboured other missense mutations. There was no difference in the frequency of *EGFR* mutations between *BRCA1*- (eight out of 17, 47%) and *BRCA2*- (three out of seven, 43%) related breast cancers (*P*=1.0). It is noteworthy that, among the 11 *BRCA1/2*-related breast cancers with *EGFR* somatic mutations, eight (72.7%) were located exclusively in the stroma (Table 2, Figure 1). Similarly, of the seven sporadic breast cancers with somatic *EGFR* mutations, four (57.1%) had mutations only in the stroma (Table 2, Figures 1 and 2). Furthermore, 57% (eight out of 14 hereditary, four out of seven sporadic) of all mutations were located in exon 20. In addition, we identified 10 somatic intronic single-nucleotide variants (ISNV) in seven of 24 (29.2%) hereditary breast cancers and nine ISNV in seven out of 48 (14.6%) sporadic breast cancers. Finally, nonsense mutations were identified in one hereditary breast cancer and two sporadic breast cancers. No in-frame deletions as reported for NSCLC were identified in either hereditary or sporadic breast cancer samples.

## DISCUSSION

Our data show that *EGFR* mutations occur at a significantly higher frequency in hereditary breast cancer compared to sporadic breast cancer (*P*=0.0079). This may not be surprising given the functional effect of *BRCA1/2* mutations: defects in BRCA1 and BRCA2 have been shown to disrupt the DNA repair mechanism, which leads to genomic instability. Despite the difference in mutation frequencies between sporadic and hereditary breast cancers, it is obvious that sporadic and heritable breast cancers both have a similarly high frequency of *EGFR* mutations in tumour stroma, and that the majority of missense mutations lie in exon 20, in contrast to those in NSCLC.

The mutations reported in gefitinib-sensitive NSCLC were located in the TK domain encoded by exons 18–21, and have been shown to increase growth factor signalling and confer susceptibility to gefitinib *in vitro* (Lynch *et al*, 2004; Paez *et al*, 2004). These data suggest that the clinical outcome after molecular targeted therapy strongly depends on acquired alterations in target genes, and, by extrapolation, perhaps germline alterations (host risk) and/or functional status of the molecular target as well. At this point, we can only speculate on the functional properties that the mutations described in our breast cancers will have on EGFR receptor signalling. The majority of variants identified lie in close proximity to the highly conserved amino-acid residues within the TK domain I to VI (Table 1). Extrapolating from other reports, it seams likely that these could affect the ATP-binding pocket and result in gain of function (Huang *et al*, 2004). Truncating mutations such as the nonsense mutations found in breast cancers are predicted to result in loss of function, and, by extrapolation from a recent report by Huang *et al* (2004) lack of responsiveness to EGFR-TKI's. Since the samples have been obtained as anonymised material, we are not able to link our results to the outcome of possible EGFR-directed therapy. Her2-*neu* expression is reported to be associated with responsiveness to therapy, especially in tamoxifen-resistant cases and it is suggested that the heterodimerisation of EGFR/Her2-*neu* might contribute. Based on our data, we did not find any evidence for a correlation between EGFR mutation status and Her2-*neu* expression (Table 1).

What the effect of *EGFR* mutations as possible targets for anticancer therapy, in the stroma on responsiveness to EGFR-TKI's, is unknown. Given our and other existing data on the tumour microenvironment (Dancey and Freidlin, 2003; Fukino *et al*, 2004), it may be postulated that the high frequencies of stromal *EGFR* mutations in sporadic and hereditary breast cancers could confound responsiveness to EGFR-TKI and may help explain interpatient variation. Thus, we suggest that future clinical trials employing molecular targeted therapy evaluate these genetic factors, not only in the traditional epithelial neoplasm but also in the surrounding tumour stroma in order to establish their role in and predictive value for interindividual variation in responses.

In summary, we have shown that *EGFR* mutations are found in a distinct, organ-specific pattern, and suggest that mutational spectra may be only one basis for prediction of response to EGFR-TKI's. Furthermore, we have demonstrated that the tumour stroma was rich in *EGFR* gene alterations compared to the epithelium. We previously reported on a model of tumour–microenviroment interaction in multistep breast carcinogenesis and pointed out the importance of mutations found exclusively in the tumour stroma (Kurose *et al*, 2001, 2002). It is acknowledged that the stroma can modulate the neoplastic epithelium by mediating invasion and progression. Therefore, it is possible that any EGFR-TK-directed therapy should consider anticancer targets in the tumour stroma as well as neoplastic epithelium, and, indeed, perhaps such TK-directed therapy in breast cancer will predominantly affect this tumour–microenvironment interaction by inhibiting invasion and progression rather than influence tumour mass.

## Figures and Tables

**Table 1:** Spectra of *EGFR* somatic mutations identified in exons 18–21 among 60 NSCLC samples, 48 sporadic breast cancers and 24 hereditary breast cancers

	**Mutation**		**Subdomain**	**Her2-*neu***
*Non-small cell lung cancer (n*=*60*)				
Sample L1	Deletion of 15 nucleotides at codons 746–750	Nucleotide 2235–2249	II	NA
Sample L2	Deletion of 15 nucleotides at codons 746–750	Nucleotide 2236–2250	II	NA
				
*Sporadic breast cancer (n*=*48*)				
Stroma				
Sample S1	T693A	ACA–GCA	I (L718)	neg.
	IVS 18+19 (g/a)			
Sample S2	IVS 18+2 (t/a)			neg.
Sample S3	A698T	GCT–ACT	I (L718)	neg.
	F712F	TTC–TTT	I (L718)	
Sample S4	IVS 18−1 (g/a)			neg.
Sample S5	IVS 18−4 (c/t)			neg.
Sample S6	IVS 18+6 (g/a)			neg.
	IVS 18+25 (g/a)			neg.
Sample S7^*^	E762K	GAA–AAA	III (E762)	pos.
Sample S8	G810D	GGC–GAC	VI (G824)	neg.
Sample S9	Y827Stop	TAC–TAA	VI (Y827)	pos.
Sample S10	IVS 19+53 (c/t)			neg.
Sample S11	IVS 20+6 (c/t)			pos.
				
Epithelium				
Sample S6	IVS 20+6 (c/t)			neg.
Sample S12	E709K	GAA–AAA	I (L718)	neg.
Sample S13	S768N	AGC–AAC	IV (V774)	NA
	W817Stop	TGG–TAG	VI (G824)	
Sample S14	R776L	CGC–CTC	IV (V774)	pos.
				
*BRCA-related breast cancer (n*=*24*)				
Stroma				
Sample H1	Q791R	CAG–CGG	V	NA
	L816P	CTC–CCC	VI (G824)	
Sample H2	L792F	CTC–CTT	V	NA
	D761N	GAT–AAT	III (E762)	
	IVS 19–15(t/c)			
Sample H3	Q821R	CAG–CGG	VI (G824)	neg.
Sample H4	N756S	AAC–AGC	III (E762)	NA
	F795F	TTC–TTT	V	
Sample H5	V742A	GTC–GCC	II (A743)	NA
Sample H6^*^	N842S	AAC–AGC	VI (N842)	neg.
Sample H7	Q821Stop	CAG–TAG	VI (G824)	NA
	V819A	GTG–GCG	VI (G824)	
Sample H8	V742A	GTC–GCC	II (A743)	neg.
Sample H16	IVS 19–3(t/c)			NA
				
Epithelium				
Sample H5	IVS 20+14 (g/a)			NA
	IVS 19+5 (g/a)			
Sample H7	I759I	ATC–ATT	III (E762)	NA
Sample H9	Q791R	CAG–GG	VI (Y827)	neg.
Sample H10	Q791R	CAG–CGG	V	pos.
	I822T	ATC–ACC	VI (G824)	
	IVS 18+19 (g/a)			
	IVS 20+14 (g/a)			
Sample H11	R748R	AGA–AGG	II (L747)	neg.
Sample H12^*^	G724S	GGC–AGC	I (G724)	NA
Sample H13	IVS 19–10 (t/c)			neg.
	IVS 20+14 (g/a)			
Sample H14	IVS 19–14 (t/c)			NA
Sample H15	IVS 20+14 (g/a)			NA

For breast cancers, both the surrounding stroma and tumour epithelium have been analysed separately (see also Figure 2). Column 2 shows affected codon with amino-acid change and corresponding base change in column 3. Column 4 indicates the corresponding TK subdomain with the closest highly conserved residue in parentheses. Her2-neu expression status is given in column 5. Samples that display a mutation in one of the highly conserved amino-acid residues are indicated by asterisk.

**Table 2:** Frequencies of *EGFR* mutations presented separately for neoplastic epithelium and the tumour stromal compartment from each case

	**Epithelium**	**Stroma**
NSCLC (*n*=60)	2 (3.3%)	Not done
Sporadic BC (*n*=48)	3 (6.3%)	4 (8.3%)
Hereditary BC (*n*=24)	3 (12.5%)	8 (33.3%)

NSCLC=non-small-cell-lung cancer; BC=breast adenocarcinoma. For NSCLC samples, the tumour stroma was not analysed separately.
